# Total hip arthroplasty, associated rehabilitation care and the COVID-19 pandemic in France

**DOI:** 10.3389/frhs.2025.1564007

**Published:** 2025-06-05

**Authors:** Carine Milcent, Pierre-Emmanuel Couralet, Saad Zbiri, Achille Tchalla

**Affiliations:** ^1^ Paris-Jourdan Sciences Economiques, French National Centre for Scientific Research (CNRS), Paris, France; ^2^ Paris School of Economics (PSE), Paris, France; ^3^ Association pour l'Utilisation des Données et l'Analyse des Systèmes de Soins (AUDASS), Paris, France; ^4^ Paris-Saclay University, Montigny-le-Bretonneux, France; ^5^ Institut d’Analyse des Systèmes de Santé (IA2S), Paris, France; ^6^ Laboratoire VieSanté - UR 24134 (Vieillissement, Fragilité, Prévention, e-Santé), Institut Ω-Health, Université de Limoges, CHU de Limoges, Limoges, France

**Keywords:** total hip arthroplasty, rehabilitation care, COVID-19 pandemic, access to healthcare, public health, health policy

## Abstract

This study investigates the Total Hip Arthroplasty (THA) activity and the associated rehabilitation care in France over the period 2013-2022, with particular attention to the COVID-19 pandemic. In 2020, scheduled THA activity was 17% lower than predicted based on the 2013–2019 trend (−19,000 THAs). In 2022, this activity was close to the pre-2019 trend, but there has been no catch-up of scheduled THAs that did not occur in 2020 and 2021. There is no obvious explanation for this absence of catch-up. In addition, in 2020, THA activity for recent trauma was only 3.1% lower than predicted based on the 2013–2019 trend. Activity in 2022 shows a return to the pre-2019 trend, without any catch-up effect, as would be expected in such a case. Finally, the proportion of scheduled THAs followed by an associated rehabilitation stay declined sharply in 2020 (−4 percentage points compared to 2019) whereas the downward trend in this proportion had previously been much slower (−10 percentage points from 2013 to 2019). These results underscore the major effect of the COVID-19 pandemic on surgical activity, though further investigation is needed to fully understand the long-term effects on patients' health and life expectancy.

## Introduction

The Coronavirus Disease 2019 (COVID-19) has given rise to a serious health crisis (1). By the end of 2020, COVID-19 had reached almost every country and affected more than 80 million people worldwide (2). COVID-19 has become a major cause of death and has caused a considerable number of additional deaths indirectly at the global, regional, and national levels. It has inevitably reduced life expectancy in many countries, with an as-yet-unknown long-term impact on morbidity (3). Word Health Organization estimates the total number of excess deaths attributable to COVID-19, both directly and indirectly, to be at least 3 million in 2020 (4). Overall, the COVID-19 pandemic has caused hundreds of millions of cases and several million deaths worldwide (2).

Public administrations have had to work under uncertainty and make difficult choices in response to the health, economic, and social challenges raised by the COVID-19 virus (5). Governments have reacted quickly, adopting extensive policies and institutional programs to control the spread of the virus and limit the strain on health systems (6). By April 2020, over half of the global population was under strict containment measures on an unprecedented scale (7). These strong containment measures managed the intensity of the first wave of the epidemic; they were gradually removed from June (8). However, the application of strict political decisions to the entire population could have negative human, health, economic, and social consequences.

The COVID-19 pandemic has caused significant and persistent disruption of healthcare services (9). Almost all countries affected by COVID-19 report one or more disruptions to essential medical services (10). The most common causes of interruption or reduction of health services include health personnel-related reasons such as the reassignment of a large proportion of health staff within the health system, disruption of supply chains limiting the availability of essential medicines, diagnostics, and necessary personal protective equipment, and the cancellation of planned prevention and treatment services (11).

In particular, COVID-19 government policy measures resulted in hospitals having to extensively reorganize their various healthcare services (12). Indeed, many non-COVID-19 related hospitalizations, whether urgent or non-urgent, have decreased significantly (13). Regarding patient demand, COVID-19 public policy measures may have also impacted patient healthcare-seeking behaviour, due especially to recommendations to stay home, fear of exposure and infection by COVID-19, and family or social isolation (6). However, the absence or delay of hospital admissions for a number of severe medical conditions can have serious consequences as it reduces the opportunity to provide effective care and improve outcomes for patients.

Over the past few decades, the number of orthopaedic surgical procedures has steadily increased worldwide (14, 15). This rise is driven by several factors, including advances in biomaterials and surgical techniques, the development of imaging technologies, improved perioperative care, and patients' growing desire to age in good health with minimal functional impairment. Among the most significant innovations is Total Hip Arthroplasty (THA), which provides substantial functional recovery and, most importantly, lasting pain relief, despite the persistence of some risks (16). In France, as in other high-income countries, the demand for orthopaedic surgery, including THA, continues to rise, fuelled by an ageing and expanding population (15).

The aim of this study was to examine and discuss the changes in inpatient admissions for THA and related rehabilitation care in France from 2013 to 2022, with particular regard to COVID-19 policy responses. Unlike previous studies that have focused on specific regions or smaller sample sizes, our study offers a nationwide perspective, using a comprehensive analysis of exhaustive national data from all French hospitals over a 10-year period, and provides valuable insights into the evolution of THA activity and associated rehabilitation prior to, throughout, and following the COVID-19 pandemic.

### Study on patients hospitalized for THA

We conducted a retrospective, population-based study of patients admitted for THA in all French hospitals from 2013 to 2022. Data were obtained from French National Hospital Discharge Diagnosis Databases (PMSI). The study population was selected, in accordance with the literature, using the French Diagnosis Related Groups (DRG) 08C47 for unscheduled THA and 08C48 for scheduled THA. DRGs are assigned to stays on the basis of (i) the codes of the International Classification of Diseases, 10th revision (ICD-10), registered as the main diagnosis, and (ii) the codes for medical procedures performed during the stay [French Common Classification of Medical Procedures (codes CCAM)] (17). All patients admitted for THA in all French hospitals between 2013 and 2022, as recorded in the national hospital database (PMSI), were included. This large population of THA patients during the study period provided a comprehensive and statistically robust sample.

The data were analyzed using Stata software (version 17). In the following analyses, we estimate the changes in the number of inpatients, in total and per hospital, for scheduled and unscheduled THAs and associated rehabilitation stays, among the study population in France, according to the intensity of the health crisis. Where relevant, we also examine the distribution of these admissions by patient age and gender, and by activity of the admitting hospital.

These variations in hospital admissions are represented graphically and are calculated using the years 2013–2019 as a reference for expected trends in THA admissions and rehabilitation stays, while considering the different periods of the years 2020–2022, corresponding to the different phases during and after the COVID-19 health crisis. We assume that, without the COVID-19 pandemic, the distribution of admissions in 2020–2022 should have followed the same pattern as in the years 2013–2019. Statistical significance is indicated by the 95% Confidence Intervals (CIs) and *p*-values of the t-tests (18).

In accordance with French law, the study was exempt from informed consent and ethical approval requirements because the dataset, based on routinely collected administrative data, did not contain any patient-identifiable information (19). The data were accessed for research purposes from the beginning of July 2023.

### Scheduled THAs: significant fall in activity in 2020 without any subsequent catch-up

**Figure 1:**
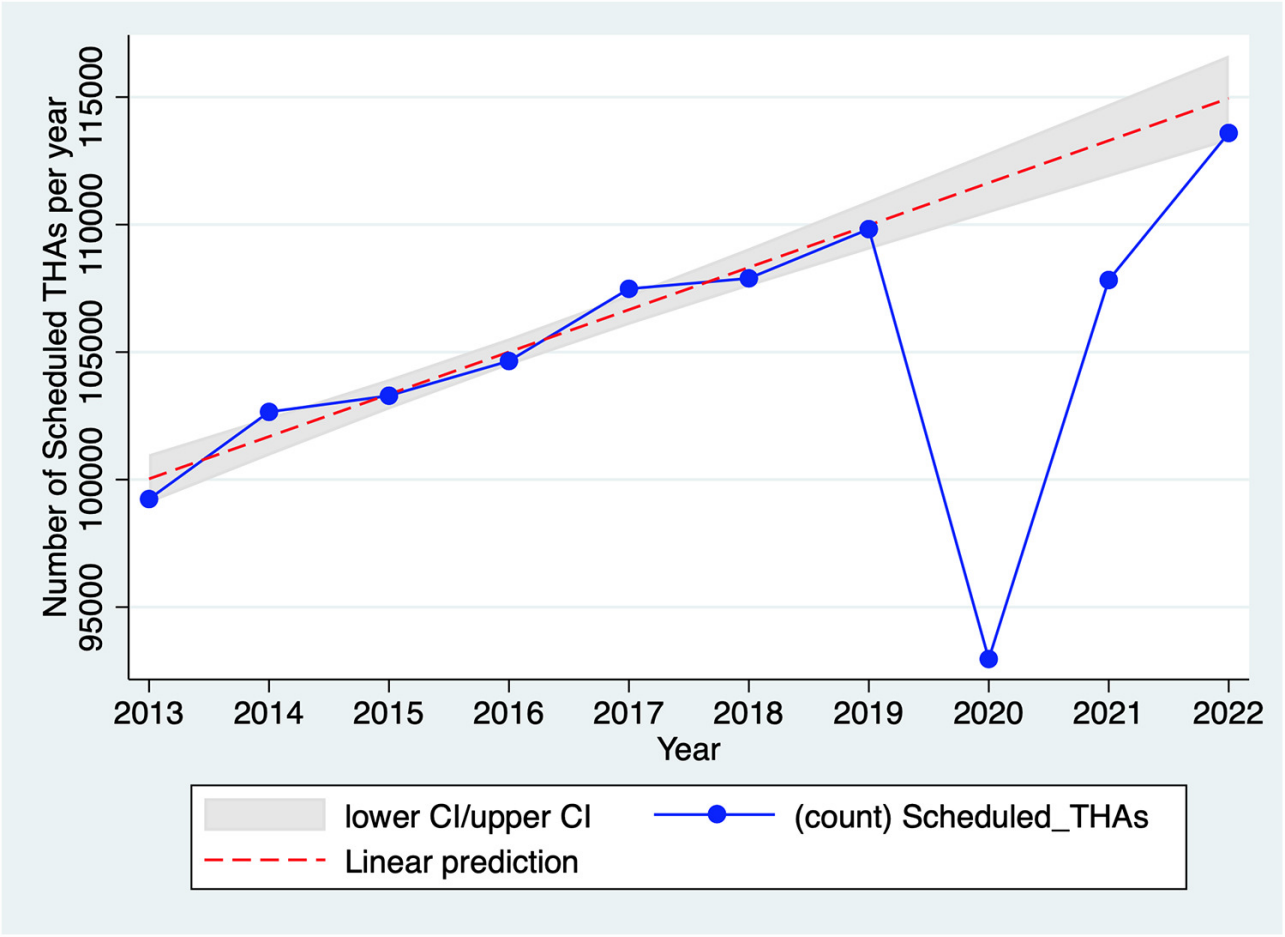
Evolution of the total number of scheduled THAs in France, 2013–2022. Source: PMSI data, 2013–2022, France (all). The *x*-axis represents the year, the *y*-axis represents the total number of scheduled THAs. The linear prediction represents the yearly trend of scheduled THAs including 95% confidence interval (CI) for the reference trend (years 2013–2019).

**Figure 2:**
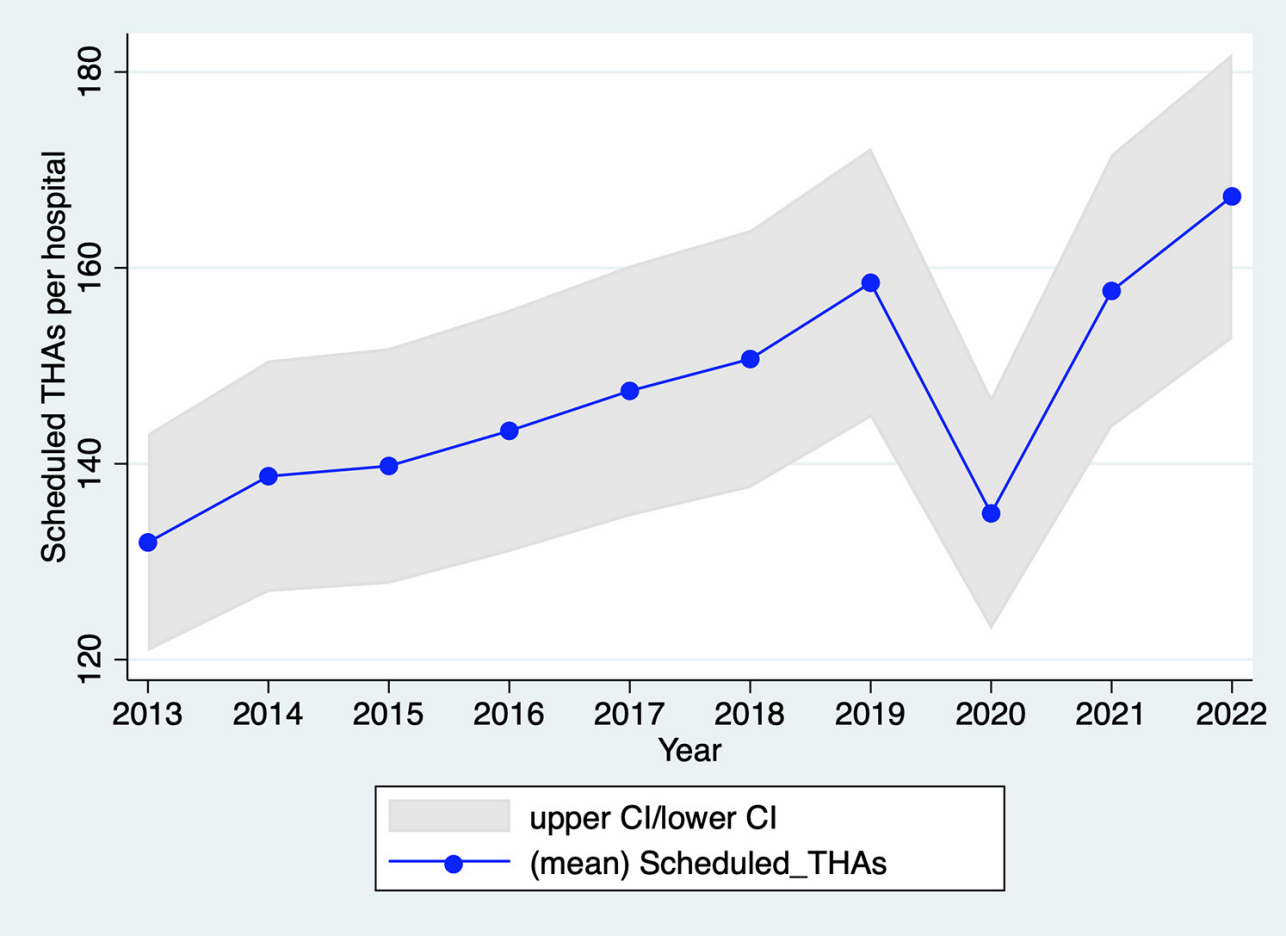
Evolution of the number of scheduled THAs per hospital in France, 2013–2022. Source: PMSI data, 2013–2022, France (all). The *x*-axis represents the year, the *y*-axis represents the mean of scheduled THAs per hospital including 95% confidence interval (CI).

In 2020, the number of scheduled THAs was 15% lower than in 2019, whereas the average annual growth rate for the period 2013–2019 was 1.7% (Figure 1). There were 16,931 fewer scheduled THAs in 2020 than in 2019 (93,270 vs. 110,701); this difference rises to 18,755 (−16.7%) when considering the trend over the 2013–2019 period. This number of scheduled THAs in 2020 is significantly lower than the reference annual trend of scheduled THAs (years 2013–2019), both overall (*p* < 0.01) (Figure 1) and by hospital (*p* < 0.01) (Figure 2). These results remain significant even after adjustment for age, sex, and comorbidities (upon request). As shown in Supplementary Table A1 in Appendix S1, the decline in scheduled THA activity is concentrated in the months of containment, i.e., March (−43% compared with March 2019), April (−97%), May (−65%), and, to a lesser extent, November (−17%) and December (−11%). It should be noted that the first three months correspond to the period of the first strict containment, during which all non-emergency surgical procedures were rescheduled, while the latter 2 months correspond to a second, lighter containment.

As per Figure 1, scheduled THA activity in 2021 did not reach the 2019 level, while in 2022, this activity returned to the pre-2019 trend. However, there was no catch-up of scheduled THAs that did not occur in 2020 and 2021. In 2021, scheduled THA activity remained 1.8% lower than in 2019 (−4.8% considering the 2013–2019 trend), due to a low number of THAs in April (−14% compared with 2019) and May (−20%), corresponding to the third (and final) containment phase. In 2022, scheduled THA activity exceeded that of 2019 (114,621 vs. 110,701) but remained slightly below the projected activity based on the 2013–2019 trend (116,493).

Finally, analyses of scheduled THA activity considered the age of patients (Supplementary Tables A1, A2 in Appendix S2). In 2022, the average age of patients receiving a scheduled THA was exactly 70 years. The median age of this patient population was 71, and the ages defining the limits of the first and third quartiles were 63 and 78 (see Supplementary Table A1 in Appendix S2). This distribution is presented in Supplementary Graph A1 in Appendix S2. It has changed slightly over the period 2013–2022, with notable trends including: (i) a slow increase in the average age (+0.8 years from 2013 to 2022) and median age (from 70 to 71 years) of patients; and (ii) an increase in the proportion of patients aged 70–74 (15.3% in 2013 and 20.5% in 2022; see Supplementary Table A2 in Appendix S2). In 2022, only 5% of patients are aged under 50 and only 2% are aged 90 or over. Finally, the distribution of scheduled THAs in 2020 was not significantly different from those in other years, indicating that the THAs that were postponed did not disproportionately affect any specific age group.

In contrast, the proportion of men among patients for scheduled THA has been lower or equal to the previous year in every year except 2020 (45% male patients compared with 44.6% in 2019), which suggests that postponed THAs in that year were slightly more likely to involve women than men (see Supplementary Graph A2 in Appendix S2).

### Unscheduled THAs: temporary and predictable fall in activity in 2020

**Figure 3:**
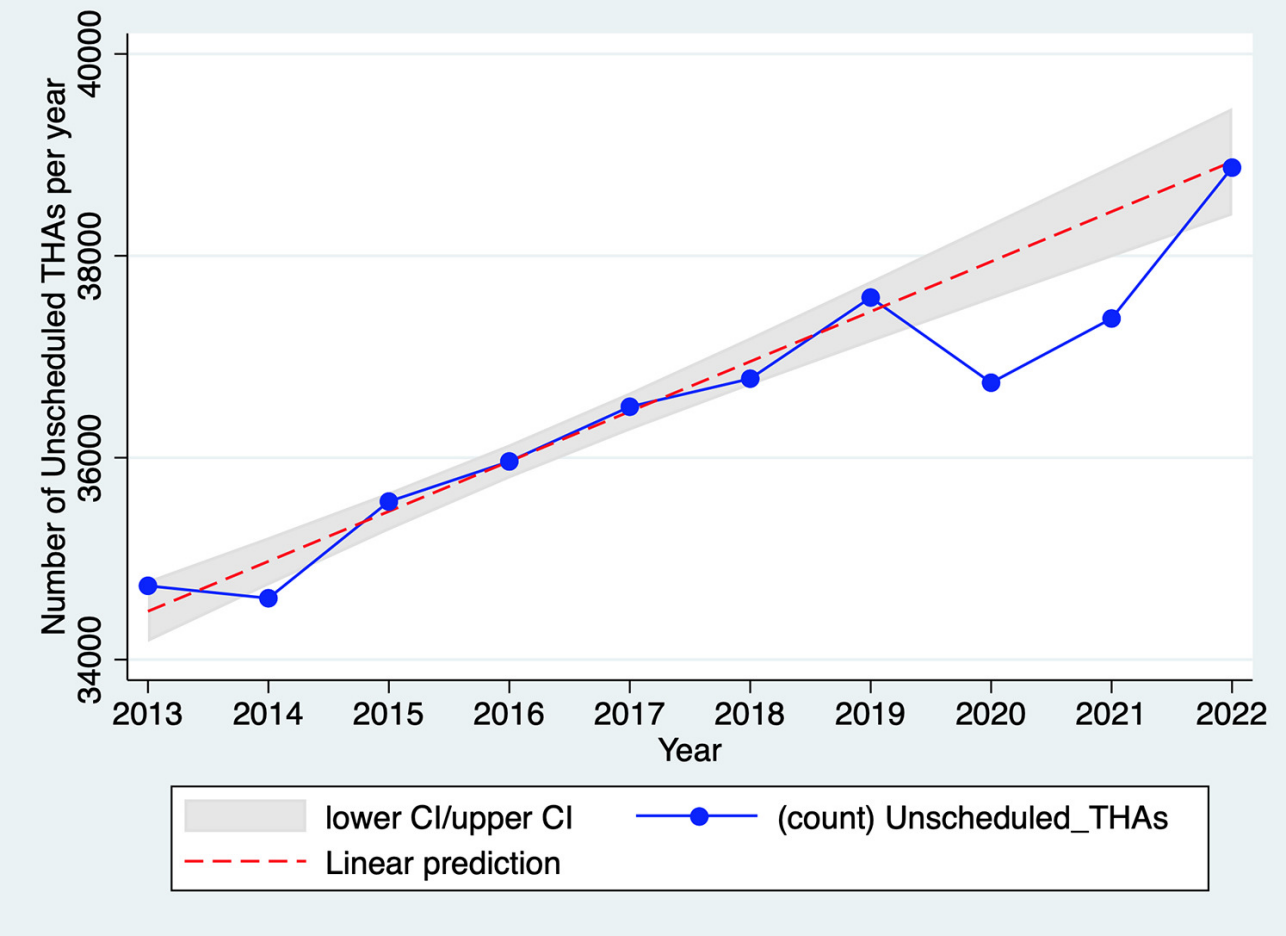
Evolution of the total number of unscheduled THAs in France, 2013–2022. Source: PMSI data, 2013–2022, France (all). The *x*-axis represents the year, the *y*-axis represents the total number of unscheduled THAs. The linear prediction represents the yearly trend of unscheduled THAs including 95% confidence interval (CI) for the reference trend (years 2013–2019).

**Figure 4:**
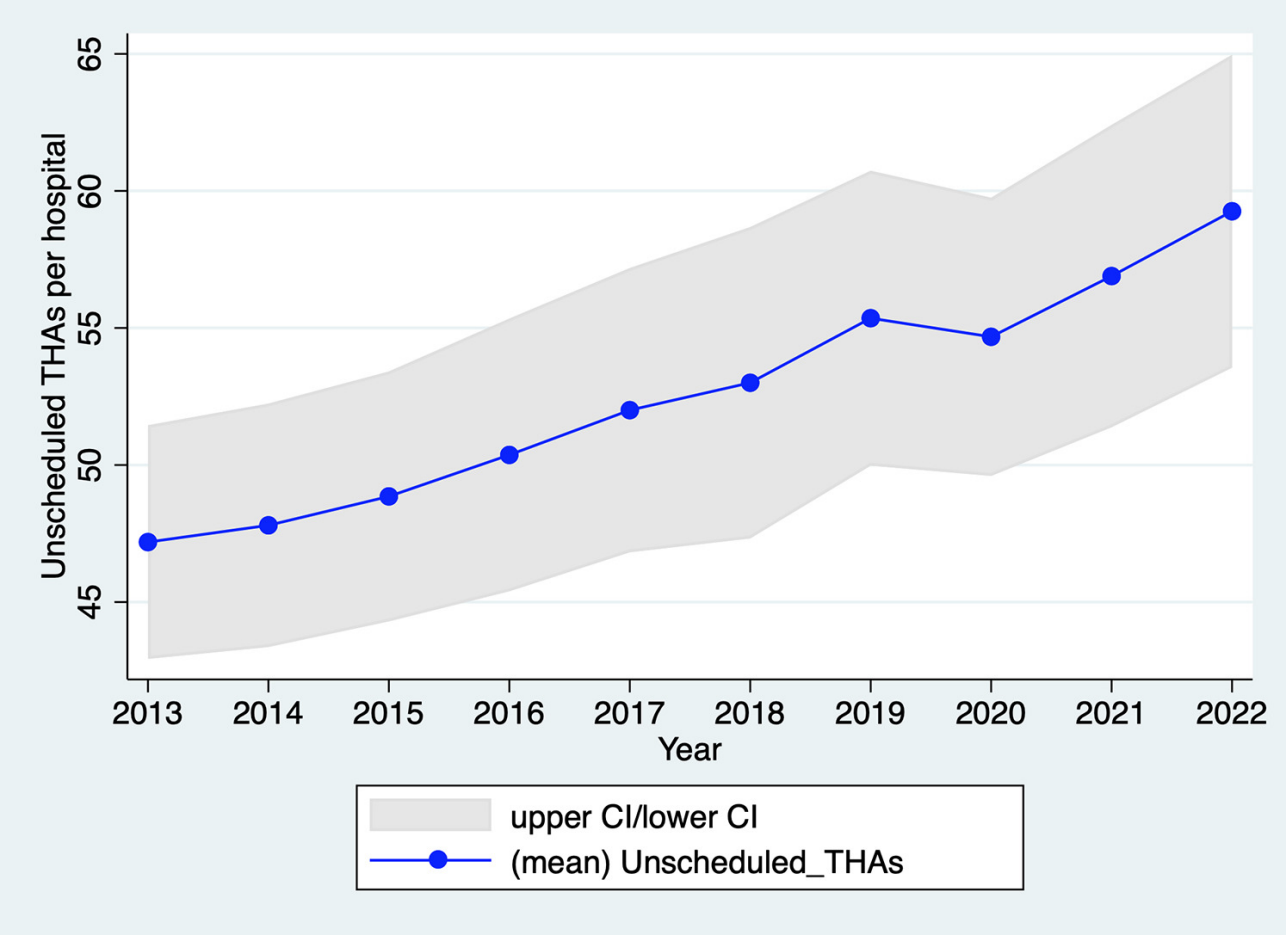
Evolution of the number of unscheduled THAs per hospital in France, 2013–2022. Source: PMSI data, 2013–2022, France (all). The *x*-axis represents the year, the *y*-axis represents the mean of unscheduled THAs per hospital including 95% confidence interval (CI).

In 2020, THA activity for recent trauma was only 2.3% lower than in 2019 (37,419 vs. 38,295); the decrease is 3.1% when considering the average annual growth rate of 1.4% over the period 2013–2019 (Figure 3). This number of unscheduled THAs in 2020 is not significantly lower than the reference annual trend of unscheduled THAs (years 2013–2019), both overall (*p* > 0.05) (Figure 3) and by hospital (*p* > 0.05) (Figure 4). These results remain not significant even after adjustment for age, sex, and comorbidities (upon request). As shown in Supplementary Table A2 in Appendix S1, the fall in unscheduled THA activity is concentrated in the months of containment (−16% in April compared with the same month in 2019; −11% in May) but it remains limited compared to the fall in scheduled THA activity. Due to their urgent nature, these surgical procedures are unlikely to be rescheduled. As a result, the proportion of unscheduled THAs among all THAs rose to 28.5% in 2020 (i.e., the fall in scheduled THAs was greater than that in unscheduled THAs), whereas it was lower and stable in the other years of the study period (25.7% on average, with a standard deviation of 0.2 percentage points; see Supplementary Table A3 in Appendix S1).

As per Figure 3, unscheduled THA activity in 2021 was still slightly below its 2019 level, while in 2022, activity returned to the pre-2019 trend. In 2021, activity was only 0.5% lower than in 2019 (−2.7% when considering the 2013–2019 trend). In 2022, activity exceeded that of 2019 (39,599 vs. 37,419) and reached the level predicted by the linear progression observed in 2013–2019. Therefore, there was no catch-up of unscheduled THAs not performed in 2020.

### Rehabilitation stays following scheduled THAs in 2020

**Figure 5:**
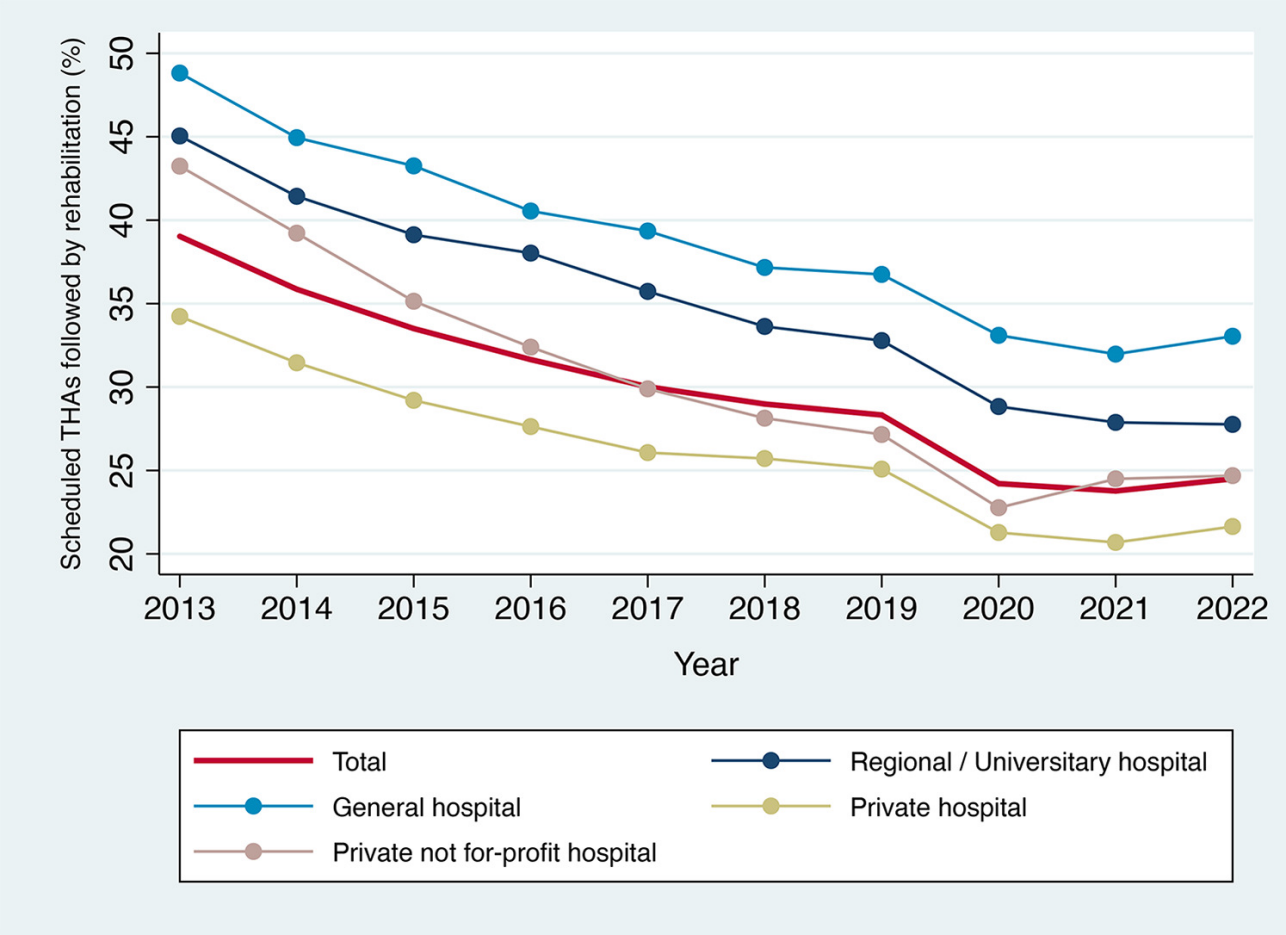
Proportion of scheduled THAs followed by a rehabilitation stay in France, by type of hospital, 2013–2022. Source: PMSI data, 2013–2022, France (mainland). The *x*-axis represents the year, the *y*-axis represents the percent of scheduled THAs followed by rehabilitation.

The proportion of scheduled THAs followed by an associated rehabilitation stay fell sharply in 2020 (−4 percentage points compared with 2019) whereas the downward trend in this proportion had previously been much slower (−10 percentage points in 6 years, from 2013 to 2019) (Figure 5). Since 2020, the proportion of rehabilitation discharges has remained fairly stable at just under 25%.

## Discussion and implications for public health policy

There is no obvious explanation for the lack of catch-up of scheduled THAs that did not take place in 2020. Factors that may play a role include: (i) premature death linked to COVID-19 and its consequences for a proportion of elderly patients who would otherwise have benefited from THA. This factor is difficult to quantify, but it cannot explain the entire drop in the number of scheduled THAs: 17% of people who should have had a THA implanted in 2020 would have had to die to explain the drop in the number of THAs without catching up, which is more than the excess mortality linked to COVID-19 in the highest-risk groups (2–4); (ii) postponement to a later date (after 2022), or even cancellation, of scheduled THAs for which there was little need (so-called “comfort” THAs, but this concept remains to be defined) due to changes in patient preferences (greater fear of attending hospitals and a reassessment of the cost/benefit ratio of the surgery) (20); and (iii) saturation of the supply of scheduled THAs: perhaps the capacity of hospital facilities enabled them to recover the level of activity corresponding to the 2013–2019 trend, but not to exceed it in order to catch up on deprogrammed THAs (see Supplementary Graph A3 in Appendix S2) (11).

As with scheduled THAs, there was no catch-up of unscheduled THAs not performed in 2020, which was expected. Unscheduled THAs, known as “for recent trauma”, are the result of hip fractures (due to falls, accidents) and naturally decreased during the 2020 lockdowns due to reduced movement and fewer accidents (21).

The steady decline in rehabilitation stays associated with scheduled THAs between 2013 and 2019 can likely be explained by technological innovations and improvements in care protocols and organization (22). The significant reduction in 2020 could be attributed to: (i) acceleration in the changes to organizational practices mentioned above as a result of the constraints caused by the COVID-19 pandemic (11); (ii) shortage of rehabilitation places during and after the COVID-19 pandemic (23); (iii) change in patients' preferences for rehabilitation care (i.e., greater reluctance to stay in hospitals, including for rehabilitation, in order to reduce the risk of contamination and/or to minimize the burden on healthcare staff) (23).

The study results have serious implications for clinical practice and public health policy. Our analysis shows that the containment measures during the COVID-19 pandemic were associated with large reductions in use of hospital surgical services, particularly for scheduled activities. This may impact long-term patient outcomes, such as increased pain and functional decline, as well as the overall efficiency of the healthcare system. It may also introduce potential risks, such as delayed recovery, and influence clinical decision-making and future surgical planning.

While studies conducted prior to the COVID-19 pandemic showed a steady increase in THA activity, with projections of continued growth in the coming years (14, 15), scientific literature focusing on the COVID-19 period reveals that the pandemic had a substantial negative impact on arthroplasty rates (24). Several studies have reported a significant decline in the volume of hip arthroplasties during the COVID-19 pandemic. This decrease was particularly pronounced for elective procedures, which were often postponed to prioritize COVID-19 care (25). The sharpest reductions occurred during lockdown periods, when non-urgent surgeries were widely suspended (26). This trend was also reported for other types of pathologies, with many elective surgeries being postponed or cancelled (27, 28). The large number of surgeries cancelled due to COVID-19 resulted in major financial losses for healthcare institutions and may have had a profound impact on patient outcomes. Initial results indicate a considerable loss in Quality-Adjusted Life Years (QALYs) for patients due to delayed arthroplasty surgeries (29).

Our result raises the question of patient healthcare-seeking behaviour and the potential loss of well-being, and possibly even life expectancy, for patients whose surgeries were deprogrammed., the medium-term question would be what have become of these untreated patients and what are the effects on their future health (13, 28). Another possible area of investigation is whether some of these were unnecessary procedures that the COVID-19 crisis has helped to reduce (20).

Future health policy efforts must ensure that while nationwide social restrictions are implemented to protect the population's health as a whole, they do not negatively impact patients' health. It is also important to consider the various levels of inequality that this type of policy-making can cause across the population. More comprehensive data, including socioeconomic information on patients, is needed to adequately address these questions.

## Conclusion

Based on exhaustive national data from all French hospitals, we examined in this study the changes in inpatient admissions for THA with regard to COVID-19 policy measures. We found a significant fall in scheduled THA activity in 2020 without any subsequent catch-up. This result raises concerns about the effects of delayed COVID-19 surgery on the future health and life expectancy of patients. Further research could investigate the long-term health outcomes of patients who experienced delays in THA during the COVID-19 pandemic, focusing on recovery times, complications, and overall quality of life. It would also be important to explore how the reduction in rehabilitation stays in 2020 and beyond affected patient recovery, with a particular focus on functional recovery and patient satisfaction. Finally, future studies could investigate whether the observed delays in THA activity and rehabilitation were uniform across different regions or if certain areas were more significantly impacted, which would help inform regional healthcare policies in future crises.

## Data Availability

The data analyzed in this study is subject to the following licenses/restrictions: the data underlying the results presented in the study is third party data. Restrictions apply to the availability of these data, which were used under license for this study. The data supporting this study findings are from Agence Technique d'Information Hospitalière (ATIH) Digital. The authors confirm that others can access these datasets and confirm that others would be able to access these data in the same manner as the authors. Requests to access these datasets should be directed to https://www.health-data-hub.fr.
