# Supplementary material for: Total hip arthroplasty, associated rehabilitation care and the COVID-19 pandemic in France
Source: Front Health Serv. 2025 Jun 5;5:1564007. doi: 10.3389/frhs.2025.1564007 (PMC12176836; doi:10.3389/frhs.2025.1564007)
Supplement: Supplementary file 1 [file Table1.docx]

**Supporting Information**

**S1 Appendix.** **Detailed number of THAs in 2013-2022.**

**S2 Appendix. Age and gender of patients for scheduled THAs.**

**S1 Appendix.** **Detailed number of THAs in 2013-2022.**

**Table A1.** Total annual and monthly scheduled THA activity in France, 2013-2022.

|  | **2013** | **2014** | **2015** | **2016** | **2017** | **2018** | **2019** | **2020** | **2021** | **2022** | **∆2020 -2019** | **%** |
| --- | --- | --- | --- | --- | --- | --- | --- | --- | --- | --- | --- | --- |
| January | 8 367 | 8 428 | 8 616 | 9 230 | 9 995 | 9 470 | 9 739 | 10 343 | 10 019 | 9 734 | 604 | 6% |
| February | 9 450 | 9 916 | 9 407 | 9 788 | 9 355 | 10 023 | 9 713 | 10 334 | 9 261 | 9 366 | 621 | 6% |
| March | 9 259 | 9 354 | 10 277 | 10 472 | 10 914 | 10 811 | 11 189 | 6 378 | 10 236 | 11 479 | -4 811 | -43% |
| April | 9 197 | 9 839 | 9 333 | 9 041 | 8 598 | 9 651 | 9 420 | 286 | 8 067 | 9 565 | -9 134 | -97% |
| May | 7 448 | 8 169 | 7 918 | 9 158 | 9 248 | 7 678 | 10 018 | 3 465 | 8 010 | 10 338 | -6 553 | -65% |
| June | 8 521 | 8 451 | 9 417 | 9 220 | 9 469 | 9 970 | 8 848 | 8 939 | 9 931 | 10 375 | 91 | 1% |
| July | 7 482 | 7 232 | 7 119 | 6 997 | 7 034 | 7 368 | 7 614 | 9 636 | 7 809 | 7 669 | 2 022 | 27% |
| August | 3 225 | 3 460 | 3 495 | 3 730 | 3 847 | 4 019 | 3 909 | 5 441 | 4 197 | 4 296 | 1 532 | 39% |
| September | 8 070 | 8 862 | 8 910 | 9 002 | 9 332 | 9 253 | 9 708 | 10 814 | 10 372 | 10 801 | 1 106 | 11% |
| October | 10 940 | 10 788 | 10 469 | 10 483 | 11 066 | 11 177 | 11 059 | 11 456 | 11 581 | 11 309 | 397 | 4% |
| November | 8 786 | 9 396 | 9 934 | 9 399 | 9 869 | 10 240 | 10 376 | 8 592 | 9 777 | 10 006 | -1 784 | -17% |
| December | 9 222 | 9 529 | 9 101 | 8 881 | 9 543 | 9 102 | 9 108 | 8 086 | 9 425 | 9 683 | -1 022 | -11% |
| **Total** | **99 967** | **103 424** | **103 996** | **105 401** | **108 270** | **108 762** | **110 701** | **93 770** | **108 685** | **114 621** | **-16 931** | **-15.3%** |
| Prediction* | 100 737 | 102 421 | 104 105 | 105 789 | 107 473 | 109 157 | 110 841 | 112 525 | 114 209 | 115 893 |  |  |

Source: PMSI data, 2013-2022, France (all).

Note: The prediction (*) is based on the 2013-2019 time period, extended to 2020-2022.

**Table A2.** Total annual and monthly unscheduled THA activity in France, 2013-2022.

|  | **2013** | **2014** | **2015** | **2016** | **2017** | **2018** | **2019** | **2020** | **2021** | **2022** | **∆2020 -2019** | **%** |
| --- | --- | --- | --- | --- | --- | --- | --- | --- | --- | --- | --- | --- |
| January | 3 133 | 3 035 | 3 089 | 2 859 | 3 274 | 3 248 | 3 409 | 3 423 | 3 082 | 3 235 | 14 | 0% |
| February | 2 818 | 2 790 | 2 941 | 2 959 | 2 993 | 2 972 | 3 097 | 3 000 | 3 058 | 3 164 | -97 | -3% |
| March | 2 996 | 2 940 | 3 166 | 3 192 | 3 228 | 3 335 | 3 118 | 3 204 | 3 361 | 3 395 | 86 | 3% |
| April | 2 911 | 3 001 | 3 101 | 2 934 | 2 876 | 2 895 | 3 120 | 2 629 | 3 218 | 3 145 | -491 | -16% |
| May | 3 055 | 2 729 | 2 797 | 3 135 | 3 137 | 3 233 | 3 156 | 2 801 | 2 981 | 3 320 | -355 | -11% |
| June | 2 711 | 2 842 | 3 070 | 3 020 | 3 031 | 2 957 | 2 929 | 3 245 | 3 070 | 3 274 | 316 | 11% |
| July | 3 008 | 3 018 | 3 136 | 2 884 | 2 948 | 3 144 | 3 463 | 3 247 | 3 165 | 3 154 | -216 | -6% |
| August | 2 818 | 2 825 | 2 842 | 3 157 | 3 058 | 2 967 | 3 062 | 2 948 | 3 165 | 3 227 | -114 | -4% |
| September | 2 860 | 2 938 | 2 949 | 2 940 | 2 975 | 2 966 | 2 959 | 3 270 | 3 161 | 3 242 | 311 | 11% |
| October | 3 044 | 3 042 | 3 015 | 3 100 | 3 207 | 3 379 | 3 478 | 3 399 | 3 147 | 3 359 | -79 | -2% |
| November | 2 771 | 2 797 | 2 883 | 3 081 | 3 190 | 3 158 | 3 056 | 2 964 | 3 173 | 3 330 | -92 | -3% |
| December | 3 141 | 3 210 | 3 181 | 3 237 | 3 177 | 3 159 | 3 448 | 3 289 | 3 511 | 3 754 | -159 | -5% |
| **Total** | **35 266** | **35 167** | **36 170** | **36 498** | **37 094** | **37 413** | **38 295** | **37 419** | **38 092** | **39 599** | **-876** | **-2.3%** |
| Prediction* | 35 004 | 35 522 | 36 040 | 36 558 | 37 076 | 37 594 | 38 112 | 38 630 | 39 148 | 39 666 |  |  |

Source: PMSI data, 2013-2022, France (all).

Note: The prediction (*) is based on the 2013-2019 time period, extended to 2020-2022.

**Table A3.** Total annual THA activity in France, 2013-2022.

|  | **C48** | ***∆C48*** | **C47** | ***∆C47*** | **Total** | **C48/Total** |
| --- | --- | --- | --- | --- | --- | --- |
| 2013 | 99 967 |  | 35 266 |  | 135 233 | 73.9% |
| 2014 | 103 424 | *3.5%* | 35 167 | *-0.3%* | 138 591 | 74.6% |
| 2015 | 103 996 | *0.6%* | 36 170 | *2.9%* | 140 166 | 74.2% |
| 2016 | 105 401 | *1.4%* | 36 498 | *0.9%* | 141 899 | 74.3% |
| 2017 | 108 270 | *2.7%* | 37 094 | *1.6%* | 145 364 | 74.5% |
| 2018 | 108 762 | *0.5%* | 37 413 | *0.9%* | 146 175 | 74.4% |
| 2019 | 110 701 | *1.8%* | 38 295 | *2.4%* | 148 996 | 74.3% |
| 2020 | 93 770 | *-15.3%* | 37 419 | *-2.3%* | 131 189 | 71.5% |
| 2021 | 108 685 | *15.9%* | 38 092 | *1.8%* | 146 777 | 74.0% |
| 2022 | 114 621 | *5.5%* | 39 599 | *4.0%* | 154 220 | 74.3% |

Source: PMSI data, 2013-2022, France (all).

Note: C48 corresponds to scheduled THA, C47 corresponds to unscheduled THA.

**S2 Appendix. Age and gender of patients for scheduled THAs.**

**Table A1.** Distribution of ages of patients with a scheduled THA, 2013-2022.

|  | **Mean** | **p10** | **p25** | **Median (p50)** | **p75** | **p90** | ***N*** |
| --- | --- | --- | --- | --- | --- | --- | --- |
| 2013 | **69.2** | 54 | 62 | **70** | 78 | 83 | *99 238* |
| 2014 | **69.2** | 54 | 62 | **70** | 78 | 83 | *102 656* |
| 2015 | **69.4** | 54 | 62 | **70** | 78 | 83 | *103 291* |
| 2016 | **69.3** | 54 | 62 | **70** | 78 | 83 | *104 651* |
| 2017 | **69.4** | 54 | 63 | **70** | 78 | 83 | *107 483* |
| 2018 | **69.5** | 54 | 63 | **70** | 78 | 83 | *107 893* |
| 2019 | **69.7** | 55 | 63 | **71** | 78 | 84 | *109 824* |
| 2020 | **69.6** | 55 | 63 | **71** | 77 | 84 | *92 966* |
| 2021 | **69.8** | 55 | 63 | **71** | 78 | 84 | *107 829* |
| 2022 | **70.0** | 55 | 63 | **71** | 78 | 84 | *113 593* |

Source: PMSI data, 2013-2022, France (mainland).

**Table A2.** Distribution of age groups of patients with a scheduled THA, 2013-2022.

|  | **<50** | **[50-59]** | **[60-64]** | **[65-69]** | **[70-74]** | **[75-79]** | **[80-84]** | **[85-89]** | **90+** | ***N*** |
| --- | --- | --- | --- | --- | --- | --- | --- | --- | --- | --- |
| 2013 | 5.7% | 13.5% | 12.9% | 16.1% | 15.3% | 16.5% | 13.0% | 5.8% | 1.3% | *99 238* |
| 2014 | 5.6% | 13.1% | 12.5% | 17.0% | 15.1% | 16.5% | 13.1% | 5.7% | 1.4% | *102 656* |
| 2015 | 5.5% | 12.9% | 12.1% | 17.8% | 15.1% | 16.1% | 13.0% | 6.1% | 1.5% | *103 291* |
| 2016 | 5.5% | 13.0% | 11.9% | 18.0% | 16.0% | 15.4% | 12.7% | 6.2% | 1.4% | *104 651* |
| 2017 | 5.4% | 12.7% | 11.7% | 17.7% | 17.3% | 14.8% | 12.4% | 6.4% | 1.5% | *107 483* |
| 2018 | 5.4% | 12.5% | 11.7% | 17.0% | 18.4% | 14.8% | 12.3% | 6.4% | 1.5% | *107 893* |
| 2019 | 5.2% | 12.1% | 11.4% | 16.5% | 19.4% | 14.9% | 12.3% | 6.7% | 1.7% | *109 824* |
| 2020 | 5.2% | 12.4% | 11.6% | 16.1% | 20.4% | 14.5% | 11.8% | 6.4% | 1.7% | *92 966* |
| 2021 | 5.1% | 12.3% | 11.1% | 15.6% | 20.6% | 15.4% | 11.6% | 6.4% | 1.8% | *107 829* |
| 2022 | 4.6% | 12.0% | 11.0% | 15.6% | 20.5% | 17.2% | 11.1% | 6.3% | 1.7% | *113 593* |

Source: PMSI data, 2013-2022, France (mainland).

**Graph A1.** Distribution of scheduled THAs according to patient age, 2022.

Source: PMSI data, 2022, France (mainland).

**Graph A2.** Distribution of age groups of patients with a scheduled THA according to men proportion, 2022.

Source: PMSI data, 2022, France (mainland).

**Graph A3.** Distribution of hospitals performing scheduled THAs according to their level of activity, 2013-2022.

Source: PMSI data, 2013-2022, France (mainland).
